# Supplementary material for: Kinetics of Serological Response in Patients with Severe Fever with Thrombocytopenia Syndrome
Source: Viruses. 2020 Dec 25;13(1):6. doi: 10.3390/v13010006 (PMC7823500; doi:10.3390/v13010006)
Supplement: Supplementary file 1 [file viruses-13-00006-s001.pdf]

Supplementary Table 1. Detailed clinical characteristics of the three deceased patients

**Characteristics**	**Died patient 1**	**Died patient 2**	**Died patient 3**
**Age (years)**	66	50	74
**Sex**	Male	Male	Female
**Season (months)**	Summer (July)	Summer (June)	Summer (June)
**Clinical characteristics**			
Fever	Yes	Yes	Yes
Tick- or chigger-bite wound	Yes	None	None
Skin rash	None	Yes	None
Bleeding	None	None	None
Myalgia	None	Yes	None
Anorexia/General weakness	None	None	Yes
Nausea/Vomiting	None	None	Yes
Abdominal pain	None	None	Yes
Diarrhea	None	None	Yes
Cough/Sputum/Dyspnea	Yes	Yes	None
Altered mental status	Yes	Yes	Yes
**Underlying diseases**			
Previously healthy	Yes	None	None
Diabetes mellitus	None	None	Yes
Solid tumor	None	None	None
Hematologic malignancy	None	None	None
Chronic liver disease	None	None	None
Chronic kidney disease	None	None	None
Chronic lung disease	None	Yes	None
Autoimmune disease	None	Yes	None
Solid organ transplantation	None	None	None
Hematopoietic stem cell transplantation	None	None	None
Immunosuppressant	None	None	None
**Treatments**			
Doxycycline	None	Yes	Yes
Ribavirin	Yes	Yes	None
Plasma exchange	Yes	Yes	Yes
Convalescent plasma therapy	None	None	Yes
Self-limiting	None	None	None
**Time of death (days)**			
Days from symptoms onset	11	7	10
Hospital days	7	2	6
**Laboratory findings**			
White blood cell (/μL)	2500	7400	1000

Neutrophil (%)	69	55	75
Lymphocyte (%)	25	36	20
Monocyte (%)	5	2	5
Hemoglobin (g/dL)	15	14	15
Platelet (× 10^3/μL)	57	53	66
BUN (mg/dL)	14	27	26
Creatinine (mg/dL)	1.72	1.55	0.73
AST (IU/L)	484	1160	124
ALT (IU/L)	197	222	69
CRP (mg/dL)	0.5	5.7	0.1
PT INR†	1.16	1.46	1.09
aPTT (seconds)†	75	113	39.6

Abbreviations: ALT, alanine aminotransferase; aPTT, activated partial thromboplastin time; AST, aspartate aminotransferase; CRP, C-reactive protein; ICU, intensive care unit; INR, international normalized ratio; PT, prothrombin time.

Supplementary Table 2. Viral load and serologic responses of the three deceased patients

Days from symptom onset*	SFTS viral load (log_{10} copies/μL)	IgG, IFA	IgM, IFA	IgG, ELISA (OD_{405})	IgM, ELISA (OD_{405})
**Died Patient 1**					
Days 1–4	N	N	N	N	N
Days 5–9	6.51	ND	10	1.03	0.51
Days 10–14	8.50	ND	ND	1.67	0.55
**Died patient 2**					
Days 1–4	N	N	N	N	N
Days 5–9	7.50	160	20	1.55	1.16
**Died patient 3**					
Days 1–4	N	N	N	N	N
Days 5–9	4.30	80	ND	4.54	0.98

Abbreviations: ELISA, enzyme-linked immunosorbent assay; IFA, immunofluorescence assay; IgG, immunoglobulin G; IgM, immunoglobulin M; N, no available data; ND, not detected; OD, optical density; SFTS, severe fever with thrombocytopenia syndrome.

*Times of death of died patient 1–3 were days 11, 7, and 10 from symptom onset, respectively.

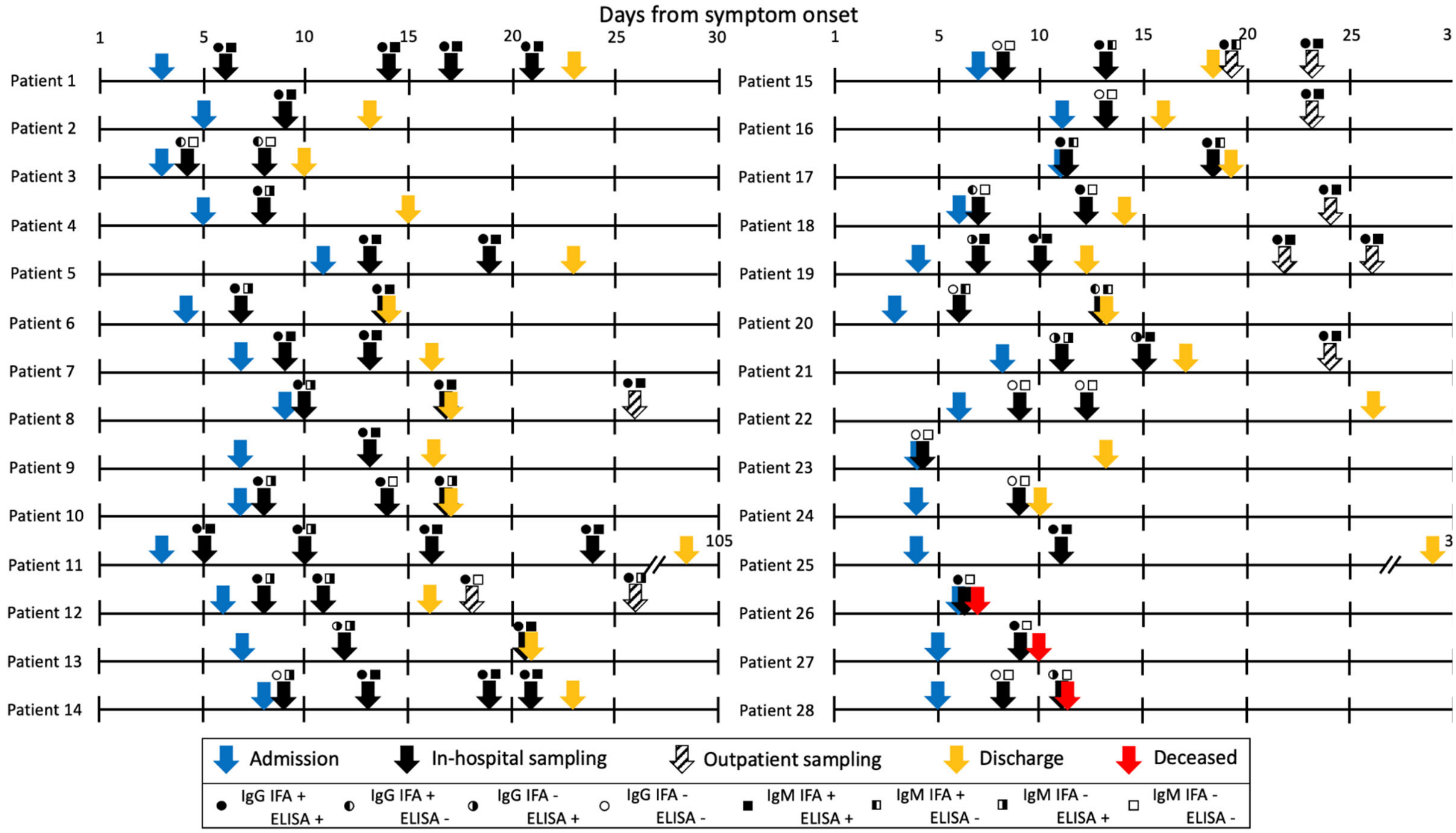

Supplementary Figure 1. Detailed information about all 28 studied patients' admission, sampling, and discharge and/or deceased times based on days from symptom onset.
